# How the master’s level is implemented in internships within master’s programmes — exploring the views of students, clinicians, and educators in midwifery and public health nursing

**DOI:** 10.1186/s12909-024-06461-4

**Published:** 2024-12-19

**Authors:** Jürgen Kasper, Turid Kristin Bigum Sundar, Lisbeth Valla, Anne Marie Lilleengen, Anne Grete Rydtun Haug, Kjersti Engen Marsdal, Iren Borgen, Victoria Telle Hjellset

**Affiliations:** 1 Faculty of Health Sciences, Department of Nursing and Health Promotion, OsloMet Metropolitan University, Oslo, Norway; 2 Division of Obstetrics and Gynaecology, Oslo University Hospital, Oslo, Norway; 3 Faculty of Health Sciences, Department of Nursing, VID Specialised University, Oslo, Norway

**Keywords:** Clinical nurse specialist, Advanced practise nursing, Evidence-based nursing, Internship, Clinical placement, Midwifery, Public health nurse

## Abstract

In the course of worldwide attempts at the academisation of professional education and the Bologna process, nursing education has become both bachelor’s and master’s programmes at colleges, polytechnics, and universities.

We investigated how a master’s level in internships is conceptionalised by the involved parties. Our focus was on Norwegian master’s education programmes in two different health professions, midwifery and public health nursing.

The study also aimed at providing a better understanding of barriers and facilitators of realising the master’s level in internships.

Using the Theory of Planned Behaviour as a theoretical and methodological framework, we conducted individual in depth interviews with representatives of all involved parties (supervisors, students, university teachers, and clinic managers). After content analysing the interview data, we held focus group meetings aiming to confirm the findings from the first part of the study.

The findings reveal a controversial debate over the necessity of a master’s level education and the challenges participants reported from their attempts to implement the master’s level into student internships. The main result is the lack of consensus on the role of internships within a master’s programme and on how students, practise supervisors, and educational institutions should collaborate on this issue. Various assumptions about the roles, accountability, and professional functions of a master’s student during an internship have been distilled into five distinct models. Instead of comparing and ranking these models, their mere coexistence is discussed as an obstacle to implementing advanced nursing practise. Recommendations are provided to help resolve this confusion.

**Supplementary Information:**

The online version contains supplementary material available at 10.1186/s12909-024-06461-4.

## Background

There is an ongoing academisation of professional educations worldwide. In Europe, the Bologna Process caused what has been called a quiet revolution in nursing education [1]. The aim has been to harmonise higher education in Europe across the professions, by implementing a common degree structure and qualification framework [1]. It ensures comparability of qualifications, facilitating student mobility and employability across member countries. Internationally, the Bologna framework shares similarities with other qualification frameworks, such as the Australian Qualifications Framework (AQF) and the UK’s Framework for Higher Education Qualifications (FHEQ). Qualifications of other systems (e.g., AQF levels 7 to 10) align with the Bologna Process, particularly regarding bachelor’s, master’s, and doctoral degrees. The training of nurses, originally located in hospital-based educational centres, moved to nursing programmes at colleges, polytechnics and universities [2]. Nevertheless, to ensure that students can learn practical skills in addition to academic training, internships are embedded in these training programmes, which can last from several months to a year. Internships are part of the graduate training curriculum and take place at relevant health facilities such as clinics or health centres. Nursing education became a bachelor’s degree, and more recently specialisations in nursing have become studies at a master’s level [2, 3]. In Norway, midwifery, and Public Health Nursing (PHN) master’s education programmes are building on a bachelor’s degree in nursing:

In 2004, the midwifery education shifted from a one-year course with a one-year internship to a full-time two-year advanced-level programme [2]. Recently, it has been further upgraded to a two-year master’s level course, consisting of 120 points under the European Credit Transfer and Accumulation System (ECTS), with 30 ECTS dedicated to thesis writing [2]. Similarly, the PHN education was once a one-year postgraduate course [4]. Under the new 2021 regulations, PHN programmes are now at at master’s level requiring 120 ECTS credits for completion, including 30 ECTS for the writing of a thesis [4]. Unlike midwifery education, which allocates half the course (60 ECTS) to internships, PHN students only need to spend 10 weeks (20 ECTS) in clinical placement [5].

Postgraduate education lays emphasis on competences in assessment, critical evaluation, and application of relevant research in clinical practise [6]. The latter includes preparing professionals to provide evidence-based care in a given individual context by considering the users’ preferences [6, 7]. There is still continuing debate about which education level is appropriate for nurses and whether academic or practise education outcomes should be prioritised [8, 9]. Although suggesting improved qualification of nurses and even improved patient outcomes, evidence for superiority of the academic nursing education is poor as it is mainly based on health care providers’ self-perception and qualitative research [8].

Master’s level education should encompass relevant theory, social learning, and the recognition of co-construction through peer communication, collaborative problem-solving, educator-facilitated critical reflections, and a learner-centred perspective [7]. These demands have significantly reshaped university coursework, emphasising the need for university teachers with advanced qualifications. However, advanced learning goals are essential not only for theoretical education but also for internships within master’s programmes. National education guidelines lack specificity regarding learning goals for the internships, leaving the standards for master’s level training during the practical study unclear [4–6]. In Norway, as in many EU countries, university teachers intermittently oversee students during internships [2]. They are expected to ensure students acquire essential clinical skills and academic competencies [2, 10]. In addition, an assigned midwife or PHN supervisor at the practise site provides daily follow-up and is primarily responsible for the student’s internship training and assessment [9]. Currently, many of these practise supervisors do not have a master’s level education themselves.

In view of this situation, we sought to acquire an understanding of how master’s level is conceptualised by those involved in arranging internships. We also aimed to understand how the master’s level is implemented in clinical practise and which kinds of barriers and facilitators the involved parties meet in the attempt to achieve the master’s level in internships.

## Methods

### Design

The study employed a qualitative design, utilising in depth interviews and subsequent focus groups, conducted separately, in Norwegian public health nurse-led municipal healthcare and midwifery services. In Norway, these services are found in both the municipal and specialist health care sectors. The individual interviews in the first part of the study aimed to gather attitudes, concerns, preferences, and ideas about efforts to achieve a master’s level during internships in these environments. The focus group interviews in the second part of the study were intended to validate and discuss findings from the initial phase of the study.

The theory of planned behaviour (TPB) was used for both the data collection and the analysis of the findings [14]. TPB is relevant for analysing behaviour by examining its underlying reasons; in this context, the target behaviour is “implementing the master’s level into internship training”. TPB explains the components involved in the internal process that prepares a behaviour, which include expectations about: 1. potential outcomes of the target behaviour (attitudes), 2. the attitudes of important others regarding the target behaviour (subjective social norms), and 3. individual behaviour control [13]. According to TPB, the realisation or implementation of a particular behaviour depends on the presence of all three components. For example, even if a person holds positive attitudes and expects positive attitudes from important others, a particular behaviour might not be implemented if the person lacks confidence in their own behaviour control. Many studies, especially those focusing on health-related behaviour, have demonstrated the usefulness and validity of TPB as a model to predict behaviour and explain variability at a group level [14]. TPB seems to be a suitable model to organise new knowledge on factors involved in the motivation to carry out behaviour related to implementing the master’s level in internships. Following our rationale the theory framework would guide the interviewer’s awareness for all types of factors potentially affecting the target behaviour.

Recruitment methods, data collection, and content analyses were based on practicality rather than adhering to a specific qualitative approach. To ensure the trustworthiness of every phase of the analysis process, including preparation, organisation, and reporting of results, we used Elo’s checklist for research-based reporting of content analyses [11].

### Sample

The sample included representatives with sufficient professional experience from all parties involved in providing internship training: supervisors, students, university teachers, and clinic managers. These individuals were recruited from hospitals, higher education institutions, and municipal children’s health clinics across Norway. Recruitment was organised consecutively, using a convenience sampling approach through existing networks. We stopped recruiting once we achieved a balanced distribution among the representative groups and considered the collected information to be saturated after conducting interviews [12]. Participants in the focus group interviews were partly from the first study phase and partly newly recruited using the same strategy as in the initial part of the study. Notably, having a master’s degree was not a selection criterion, as many supervisors do not hold such a degree.

### Data collection (in depth interviews & focus group interviews)

The interview guide was created through an iterative discussion process within the research group and piloted using roleplays in the research group including colleagues not directly involved in the study as interviewees. The interview had two main objectives. First, it aimed to explore how participants individually understand and define the master’s level in the context of internships, considering their professional roles and perspectives. Second, it sought to identify barriers and facilitators to carrying out behaviours involved in the implementation of the master’s level in internship training. The investigation of the latter objective was structured by the framework provided by the TPB. The structure of the theory was used in the interview guide as a template to consider all relevant components of action planning.

The individual interviews lasted about 60 min and were conducted pairwise by a total of eight interviewers from the research group, either in physical or digital meetings. Notes were taken during and immediately after the interviews, in the prepared structured template. All interviews were audio-recorded. At the end of the interview, participants were asked whether they would also consider participating in a focus group interview later on. The focus group interviews were planned separately for the two professions. Additionally, we took care to assemble the groups using both newly recruited participants and some of those who had already participated in individual interviews.

We used the same interview guide for both professions and all four perspectives (students, practise supervisor, practise leader, supervisor representing the educational institution). The guide consisted of four parts:

1. We mapped the participants’ background with regard to any experiences of being involved in the conduct of internship training.
2. Participants were asked to articulate their understanding of what makes the difference when internships change to a master’s level. In this part, we collected operationalisations of the term “master’s level” applied to the specific setting of the internship. To facilitate reflection, participants were asked to report on differences between master’s level and further training, for example, with regard to the participants’ own role in the training.
3. In addition, experiences with implementing a master’s level into the arrangement of internships were collected. In this third part of the interview, TPB was used as a structure in order to focus on barriers and facilitators in all features potentially relevant to the planning of behaviour. The collected descriptions could be framed as challenges, needs, suggestions for solutions, opinions or descriptions of behaviour conduct.
4. We also collected suggestions for how an expansion of the capacity for internship trainings could be realised. This research question was included in this study, as getting more students into internships seems to represent the bottleneck regarding capacity in many health care training programmes. It was also hypothesised that there was a potential relation between the quality of the internship training on the one hand and the resources and respective capacities on the other. However, as this part of the study focused on another research aim, more detail about this part of the project will be reported elsewhere.

After analysing the individual interviews and considering the results from the study’s first part, the research group developed the interview guide for the focus groups. The focus group interviews aimed to validate the individual interview results, so participants received a summary and were prompted to discuss specific questions. The authors considered the mixed setting of the focus groups, which included various perspectives on internships, ideal for discussing the definition of the master’s level, especially concerning internships. This focus aligned with the second part of the individual interviews. Participants were encouraged to engage in discourse on this topic and try to reach consensus. The instruction aimed to elicit controversial statements rather than harmonise opinions within the group. We assumed that efforts to achieve agreement would reveal contrasts, making content analysis easier.

The focus group interviews were conducted online. The meetings lasted two hours and were moderated by two members of the project group (midwives: JK, AML IB; PHN: JK, LV, TKBS) while a third person took notes in addition to an audio recording. To be able to describe the sample, we collected personal data, such as contact data and other personal information, professional work experience, place of work, and functions of the place of work.

### Analyses (interviews, focus groups)

The interviewers documented the individual interviews in the prepared templates, while the focus group meetings were transcribed verbatim (LV, IB).

*Analysis of the individual interviews*: First, the collected notes on the documentation sheets underwent some formal editing. We assigned unique identifiers to all statements by number, profession (midwife/PHN), perspective (student/teacher/supervisor/clinical leader), and interviewer pair. We moved any incorrectly classified statements to another TPB category in the template. Next, we combined the data from individual interviews within each profession. This resulted in two documents for each profession (midwives and PHN): one detailing operationalisations of a master’s level in internships, and the other containing experiences from attempts to implement the master’s level into internships. The latter was presented in the structure of the TPB. We conducted content analyses separately for these four data pools, following these steps:

1. Reading, rephrasing, and moving to another TPB category if appropriate. This was done in dialogue with the interviewers.
2. Identification of statements carrying meaning. Statements were considered meaningful in relation to the first study question, if they provided any operationalisation of master’s level in the internships of the particular professional group. Statements not addressing the study questions were removed. Regarding the second study question, statements were considered meaningful if they described either inhibiting or facilitating factors in conducting internships on a master’s level
3. The researchers grouped similar statements into preliminary sub-categories within the three main categories given by the theory. They treated frequently appearing statements with similar meaning (redundant) in the same way as rare statements in the documentation, if not completely identical.
4. The researchers continually combined and adjusted tentative categories.
5. An intermediate level of categories (between the sub-categories and the main categories) was developed by grouping the existing sub-categories.
6. The project group discussed the category system, and they agreed upon short summaries of the midt-level-categories.

*Analysis of focus group interviews:* Analysing the focus group interviews involved an inductive process to discover both obvious and hidden patterns, arguments, or positions. Unlike the individual interviews, which focused more on specific statements, the focus group analysis utilised the information from group interactions. Responses during the conversation helped contextualise individual statements. Full transcripts of the group discussions enabled reliable identification of meaning carrying units, patterns, arguments, or integrated positions. For example, participant A’s statement would be discussed further by participants B and C, before participant A added more detail to her position. This analysis allowed for tracing sequences of statements to distil entire types of arguments.

### Ethics

The project was approved by the Norwegian Center for Research Data (NSD) with reference number: 385237.

An information and consent form were mailed to all participants in advance so they could review it and ask questions if needed. Due to the corona pandemic, the procedures for obtaining consent were simplified, allowing verbal consent to be sufficient. Conducting focus groups online presents specific challenges. Although participation doesn’t involve face-to-face contact or long-term personal involvement, the social context may lead to unintended disclosure of personal information, with participants potentially revealing more than intended. Participants were initially informed about the purpose of the interviews and the research design. They were assured of privacy in the collection, storage, and handling of data in accordance with Norwegian legislation rules [15]. Furthermore, participants were told they could withdraw at any time without providing a reason. Additionally, it was stated that those who wished could have their data deleted at any time during the process.

## Results

### Description of the sample

Between October 2020 and February 2021, a total of 26 in depth interviews were conducted, 15 with midwives and 11 with PHNs. The interviews lasted 30 to 60 min. In April 2021, eight midwives participated in a focus group interview about internship training as part of the midwifery master’s training programme. In June 2021, 5 PHNs participated in a focus group interview related to the PHN training programme. The focus group interviews lasted 120 min.

Table 1 provides an overview of the participants’ specific role in the training programmes. Due to an overwhelming positive response, recruitment was uncomplicated. None of our requests was rejected.

**Table 1:** Description of the study samples

Professional background	19 midwives	
Description of the study samples		
	12 public health nurses	
	1 other	
Region	1 Agder	
	2 Innlandet	
	18 Oslo	
	2 Troms	
	2 Trøndelag	
	1 Vestlandet	
	6 Viken	
Representative group	8 teachers	
	11 supervisors	
	6 students	
	7 leaders	
Interviews	15 individual midwifery	
	11 individual PHN	
	1 focus group midwifery	(4 of 8 participants new)
	1 focus group PHN	(2 of 5 participants new)

During the individual interviews, we collected extensive material about facilitators and barriers with regard to realising the master’s level in the internships. We also collected many concrete suggestions for solutions of the challenges met on the different sides of the endeavour. However, during the collection and analysis of these statements, it became obvious that there was both large variation and pronounced uncertainty regarding definitions and or operationalisations of what master’s level in this particular setting of the training programme (internship) would mean. Therefore, we used the additional potential of the focus group setting to broach the issue and deepen it while observing the dynamic of this discussion between the representative groups.

### Challenges related to the implementation of the master’s level in internship training

In the interviews we collected descriptions of barriers or solutions to overcome barriers. Abstracting from these diametrical modes of appearance, we structured the challenges according to the three main categories of the TBP: Attitudes, Subjective social norms, and Control believes. The analyses of the individual interviews revealed that the internal structure within the main categories given by the TPB was similar for both educational groups. Therefore, results are reported together. Where appropriate, differences between the two groups are indicated. The findings are structured according to the TPB, using three main headings:

#### Attitudes: expectations regarding the consequences of implementing the master’s level in internship training

The interviews revealed a diversity of attitudes linked to the internships in midwifery and PHN. These attitudes are categorised under three main themes:

Attitudes related to professional quality: The interviews collected attitudes potentially affecting the level of professional performance in two directions. Concerns were expressed regarding whether students would achieve sufficient practical in addition to academic skills. Other statements emphasised practise at a master’s level with a stronger focus on research as stimulating and positively impacting knowledge and competence in clinical practise.

Attitudes related to the quality of training: Similar attitudes are reflected in the discussion about the master’s level’s potential impact on the quality of the education to become a PHN or a midwife. Some consider graduates with a master’s degree less qualified to fulfil the responsible tasks in practise. They believe that this is due to a greater distance from the very subject of their professional work. Other participants argued, on the contrary, that through more reflection and the ability to update their knowledge, candidates would become better practitioners in the long term.

Attitudes concerning professional self-image vary among supervisors of internships. Some link pride in professional self-image to the traditional, experience-based approach to clinical practise and training. Others connect it with academic skills, innovation, and research-based practise. This duality is evident in both midwives and PHNs. Within the PHN group, some educators worry about losing their field’s unique character since PHN education has recently been integrated into a generic umbrella master’s programme covering 15 professions. However, many participants believe that master’s level education enhances professional self-efficacy and significantly boosts candidates’ value and employability in the job market.

According to our findings from the interviews, the aforementioned attitudes do not appear to present as homogeneous patterns attributable to the representative groups (students, practical supervisors, teachers, managers). On the contrary, they are spread across the professional borderlines.

#### Subjective social norm: mutual expectations perceived by the involved parties

This category comprises the actor’s expectations regarding expectations of others about whether the master’s level should be implemented in the conduct of internships. We found partly mutually contradicting subjective social norms amongst the parties involved in arranging and carrying out internship training for master’s students. There are expectations regarding each other’s contributions to making the internship training happen, and expectations regarding what internships in particular should be like. However, the most important finding seems to be the demanding expectations all parties face from both society and the Ministry of Education and Research regarding high degrees of professional maturity in fully graduated candidates. The latter is often defined by the level of craft skills. However, some clinical supervisors experience such ambitions as strongly interfering with the goal of developing the candidate's academic abilities. Both unmet real expectations and falsely assumed expectations might present potential barriers to the implementation of a master’s level of internships.

#### Control beliefs: expectations regarding success with attempts to implement the master’s level into student internships

According to TPB the third category of factors involved in action planning comprises the actor’s control beliefs, which is expectations regarding the actor’s own capacity of performing the action and handling it’s immediate consequences. Here, we have allocated collected suggestions for solutions to succeed with implementing the master’s level in the internship training. In the interviews, we found a strong emphasis on the competencies required for supervising internships and the resources available to support supervisors. Supervisors often feel uncertain about their responsibilities when overseeing master’s level students, especially when it comes to providing critical reflection and feedback. The traditional master-craftsman model, where students mainly learn from a role model, is considered vulnerable due to the crucial relationship between students and supervisors. Both supervisors and department heads recommend addressing these uncertainties by organising supervision seminars for pedagogical guidance and setting clear learning goals. There is also a shared desire for educational institutions to provide closer follow-up during internships. Interviewees suggested various ways to adjust or create new learning and evaluation methods to enhance the internship experience.

The teachers seem to be less concerned about the supervisors’ competence. However, they consider the quality of the cooperation and the interaction between the educational institutions and practise sites to be crucial for the students to achieve master’s level.

### A taxonomy on definitions of student internships at the master’s level (results from individual and focus group interviews)

Definitions of the master’s level within internships vary widely due to underpinning assumptions by individuals and groups of representatives. First of all, variation results from diverging ideas about which part of the study programme should develop master skills. There is no agreement amongst all participants on the assumption of a need for the master’s level in all parts of the programme, including the internship.

Next, analysis revealed a variety regarding the accountability for the programmes’ academic level. Accountability can be placed at the educational institution, in the clinic, and at the student, and can be shared. Here, we recognised voices from clinical supervisors feeling overwhelmed by the load of an assumed accountability. However, representatives from the educational institutions largely assume that the accountability for achieving a master’s level is shared between all involved parties; this includes the clinical leaders and the practise supervisors shaping the practical parts of the programme.

Third, variety was observed due to requirements for the clinical supervisors. Many of them assume that high end performance according to evidence-based practise is expected. It might seem logical that skills in critical thinking and the use of research evidence are basic requirements for clinicians supervising master’s students. However, this particular assumption can be challenged and it is not naturally accepted by all sides in our study.

As a fourth source of variety, conceptualisations of master’s level during internships differed according to the roles and functions to which the candidates were assigned. It appears to make an important difference whether the student is assumed to just practise clinical procedures or to be bearer of an essential function in the network between the educational institutions and the clinic.

A variety of ideas about the nature of internships at the master’s level coexist, resulting from the individual emphasis and composition of underpinning assumptions.

The analysis of the individual interviews and the two focus group discussions distilled five types of definitions, henceforth referred to as models (Tables 2 and 3). The first three models fit the data from both groups. The integrative model (model 4) was highlighted among the group discussing midwife education, while the agent of change model (model 5) was most prominent among the PHNs.

**Table 2:** Quotations from the focus group meetings

Quotations midwives	Quotations public health nurses
**The pure practise model**	
**Supervisor:** *Practise is where the student develops relational skills. These clinical placements are also about the student to pass approval of aptitude regardless of the master level* **Leader:** *When I was studying in Australia, bachelor students went alongside with master’s students. We did not have any different practise …* **Supervisor:** *In the internship the students need training in large numbers, there is no difference in how you handle a birth giving woman dependent on whether you are master’s student or not. It is the experience that counts* *It is at school (University) where it makes the biggest difference between bachelor and master ‘s level, just put it this way, it’s not in practise*	**Student:** *The supervisor traditionally has a role of the master in the master-apprentice model. The public health nurse student has to learn from the experience of the master. And here, practise is practise, what is trained by the student is professional practise, regardless of the level. It’s the same*
**The add-on practise model**	
**Leader:** *In master’s practise turn the supervisor has to spend the time to sit down with the student to practise critical reflection and this is demanding for the supervisor too, which shall be interested into learning about new research and stuff* **Supervisor:** *Master’s practise is placing high demands on the practise environment. Practise is expected to work evidence-based* **Student:** *Amongst other things, this implies that the supervisor has a master herself and is skilled in reflecting and supervision*	**Teacher:** … *regarding reflection … there is a huge amount of reflection in practise. But it's very much about reflection on the public health nurse's experience with the user, with themselves, and with co-operation partners. But what's missing is the next step, critical reflection* **Supervisor:** *I think there are always public health nurses who think it's exciting to work with students, but they know that it's extra work. And then you say that the manager has to make sure that the public health nurse has a little less to do*
**The meta practise model**	
**Teacher:** *I agree … physically you do the same regardless of the level of the training. But, you know, not everything you do is visible. Maybe the master’s level is about verbalizing knowledge which isn’t necessarily visible* **Supervisor:** *The clinical training is on master’s level because the trainee is prepared already at the university. This is why the student meets the same situations on another background. Of course, it is the usual daily clinical practise, but the student is by the university required to process all experience using theory and the evidence base. This makes it to become another kind of a learning process* **Supervisor:** *They are doing the same, however, it is called master’s level, as they practise abstraction in addition*	**Leader:** *But that doesn't mean that you need to arrest the supervisor out there who doesn't do it, so it's the student's wisdom then, because you actually have a lot to learn from the craftsmen out there (i.e.: practise is practise, craftsmanship must be prioritised, many questions and answers that don't come up in the education programme must be learned here). Master's preparation takes place at university* **Student:** *And then I, personally, really missed having a practise supervisor from the school who I could involve and discuss things with, because I found it difficult in practise* **Supervisor:** *I think that if you want to get practise to come to master's level, then it is to do it that way to have more academic, greater academic requirements for the written assignments, because you may not be able to do much with the practise itself at master's level, you can't vaccinate more master's like or weigh a child more like a master*
**The integrative practise model**	
**Leader:** *When it comes to master’s students’ clinical placements, we share responsibility for the student’s achievement. All are contributing to lifting the level of reflection. The university teacher will be responsible for the learning goals. The supervisor needs pedagogic skills* **Teacher:** *It’s a collaboration between the student, the clinical supervisor and the teacher around the training of clinical skills* **Teacher:** *Well, it’s not up to me to sum up, but isn’t it all about linking it to theory and reflection and everything lying between, that’s supposed to be developed? I say this is about master’s level*	**Supervisor:** *We intend the internship programme to be at master's level, but the clinic will have primary responsibility for the internship, i.e. how knowledge is translated into clinical action, and then it will be the main task of the programme to create work assignments that enable the students to use their experience through critical reflection, for example. Or that they translate theory into practise in the programme, and that's what gives them a master's level in their practise* **Teacher:** *The students should gain realistic practical experience that involves quality improvement, project work, research and practise*
**The agent of change practise model**	
	**Teacher:** *Students register learning activities in the fields: 1. regular consultations in the national programme, or what everyone gets. It goes to 2. open consultations. 3. interdisciplinary collaboration, and then 4. research, development and project work organisation and management theory are included as learning objectives. So, we need to get better at the school at operationalising it better, so that it doesn't become such a big operation* **Teacher:** *The challenge is that you get too little knowledge out of all the professional development that takes place in practise, and that's what we want to do something about when we collaborate between education and the municipality, in relation to master's and master's students, because then we can get master's theses that can lift and put research on the professional development that is already taking place* **Leader:** *So, there's an idea behind this, but when we start, it's a bit confusing and people don't quite understand what it is, but public health nurses and managers of public health nurses are positive about students, but it's obviously difficult in those municipalities that don't have a culture of systematic change and quality improvement work. And that's where we struggle a little bit*

**Table 3:** Conceptualisations of internships at the master's level

		Conceptualisations of internships at the master’s level			
⬇ Models	Is there a master’s level in internships?	Accountability for master’s level of internship training?	Role and functions during the internship		
			University	Supervisor	Student
**Pure practise**	no	university	none	practical skills training	
**Add on practise**	on top	clinical supervisor	little or none	provide academic training	knowledge transfer
**Meta practise**	implicit	university	facilitate critical reflection	practise as usual	knowledge transfer
**Integrative**	explicit	joint efforts	triangulated collaboration		
**Agent of change**	explicit	student	contribute academic skills	support quality improvement	equipped with competence on master’s level

The Table 2 shows example quotations from both focus groups. They are assigned to the five conceptual models of master’s level internships, rather than being presented in chronological order. Findings from individual interviews are not shown here, because they were administered in the analysis as notes, not as original quotations from the recordings.

## The pure practise model

According to this model, internship training remains more or less unaffected by the academic training. The curriculum during the placements is predominantly determined by the requirements and needs related to the acquisition of clinical skills. “Practise is practise, irrespective of the academic learning goals (in other parts of the programme).” This model implies a duality of theory and practise.

## The add-on practise model

This model represents the idea that the clinical supervisor is predominantly accountable for realising a master’s level during master students’ placements. This includes teaching of evidence-based practise, providing reflection, and stimulating critical thinking. This duty comes on top of the supervisor’s traditional tasks of developing practical clinical skills, and is therefore experienced by supervisors as burdensome. This model also implies that the supervisor holds at least a master’s or a higher academic level.

## The meta practise model

This model is characterised by the idea of adding a meta level to clinical practise. The “meta practise model” also states, as the “pure practise model” does, that ‘practise is practise.’ However, it adds that ‘the same is no longer the same as it is recognised and reflected by the student.’ Here, the students’ observer perspective and their role in a didactic approach are acknowledged. Although practise appears equal and even the supervisor might act in the same manner, the implicit didactic approach is different (compared to the pure practise model) due to the student’s background. This model implies the idea of a transfer of knowledge between the sections of the study programme and does not necessarily require a high academic level on the part of the clinical supervisor.

## The integrative practise model

In this model, internships are defined as cooperation between the educational institution, the clinic or respective practise, and the student. The master’s level is assumed through joint efforts and attempts to stimulate the student’s learning process on several levels (practical, theoretical, and research). The educational institution is accountable for the coordination of this collaboration. However, in this model, all sides are contributing. The model is process-oriented and integrates the resources of all parties in order to achieve the best outcomes. In contrast to the former three models, in the integrative practise model, the master’s level appears to be already implemented, while the candidate is guided through the process to achieve this level.

## The agent of change practise model

In this model, practise at a master’s level is defined by function. Unlike the integrative model, where practise is an outcome, the master’s level develops through collaboration between the candidate and professional clinical practise. The aim is to equip students with the competence needed to function at a master’s level during their internship training, rather than just achieving a certain level of knowledge and competence. With support from clinical supervisors and university teachers, the candidate acts as an agent of quality improvement. Positioned between the educational institution and the clinic, the candidate catalyses quality development and knowledge growth through small projects, truly practising at a master’s level. While this model resembles the integrative model, it is more rigorous, emphasising that positioning the candidate as an academic resource can lead to increased knowledge.

## Discussion

The current study was designed to illuminate the underpinning concepts of training master’s students during practical placements. Representatives of all parties involved in the conduct of internships during master’s trainings of midwives and Public Health Nursing (PHN) were included: students, practise supervisors, teachers and leaders at the practise workplaces.

The study provides insight into the variety of attitudes and experiences related to the conduct of internships of students of midwifery and PHN at a master’s level. It shows a partly controversial discussion about whether a master’s level is needed, and challenges met during attempts to enable students practicing master’s level during internship training. For example, some perceive the master’s level as a barrier to appropriate training of essential practical skills, while others perceive it as stimulating sustainable quality improvement in practise. The study also gained a multiplicity of suggestions to facilitate implementation of master’s level in internships.

The main result is the lack of a consensus about the role of a clinical placement in the didactic context of a master’s programme and about how students, practise and educational institutions should interact on this matter. Due to varying assumptions regarding roles, accountability, and professional functions of a master in clinical practise, definitions identified in this study are rather divergent. Through qualitative content analysis we have condensed this divergent picture in a set of five distinct models.

Importantly, the diversity displayed in the data does not give the impression of being explainable by the groups of representatives contributing to this study. Different positions present crosswise to the perspectives and roles illuminated in this study, rather than being affiliated to professional groups.

The current study relies solely on qualitative methods and does not aim to be representative or objective. The existing diversity of attitudes and ideas may not have been fully captured through the convenience recruitment strategy. However, the qualitative approach has been chosen to understand the spectrum of views on the conceptualisation of the master’s level, not to address how positions are distributed across roles, regions, or individuals. Future studies should involve larger samples to explore the distribution of attitudes and opinions identified in this study.

The order in which the methods were applied can be challenged, in particular using individual interviews before the focus group interviews. Conducting the focus groups first to inform the strategy of individual in depth interviews might have contributed to an even deeper understanding. The individual interviews could then have been used to generate narratives for an in-depth investigation of individual rationales [16, 17]. However, when we chose to start with the individual interviews, we were not yet aware of the extent of diversity amongst the underpinning definitions of the behaviour we wanted to study. This finding seemed remarkable enough to inform the focus of the group interviews. In this regard, we consider it a strength of our study design to plan the second method in order to further validate the results gained by using the first method.

### What do the findings of the current study mean in the broader context?

Theory of Planned Behaviour indicates that motivational factors influence and explain behaviour [13]. Given the diverse ways and methods of structuring master’s student internships, we asked the participants to discuss their expectations regarding the outcomes of completing a master’s degree. This approach aimed to use insights into perceived barriers to inspire the development of specific measures addressing these expectations. Our rationale was that the study could contribute to a better understanding of this often overlooked aspect of education and even lead to improvements. The study has successfully identified and organised numerous reasonable expressions of attitudes, subjective social norms, and beliefs about self-attributed control.

Some people believe that requiring a master’s level for clinical practise is unnecessary and worry that academic training might hinder the development of clinical skills. This belief could discourage supervisors from focusing on academic training. Several articles discussing trainees’ experiences with the academisation of study programmes have reported this concern about the conflict between clinical skills and academic training [2, 10, 18]. The current study also mentions another aspect of academisation: the perception that learning through professional role models is outdated. The approach of fostering learning by creating a sense of belonging in a work culture might clash with an academic training approach [19]. An academic approach would separate knowledge from the expert person, making relational learning through expert models unnecessary.

Conversely, we also encountered the opposite perspective, which values the potential impact of master’s training on sustainable quality improvement. This view is supported by others who emphasise the growing expectations for clinical educators to stay informed and up to date [2, 10].

Another extensive discussion related to concerns on the side of the clinical supervisor expecting to be forced to spend time, which is scarce, on academic activities.

The findings highlight the varying levels of professional self-efficacy and the bilateral needs for information and cooperation, which are crucial for success in master’s training. Moreover, the motivation of practise supervisors to engage in providing master’s level internship training depends on how well-equipped they feel with necessary resources. Other authors have emphasised the need for collaboration among academic staff in educational programmes [20]. The literature underscores the important role of academic institutions in training advanced practise nurses (APN) or advanced midwife practitioners (AMP), who serve as role models for postgraduate nurses and midwives, especially during internship placements [20, 21].

The examples provided show that the TPB is suitable for mapping the motivational structure of actions attempting to achieve a master’s level in internships and that these findings are reasonable within the literature context. However, the most significant discovery of our study emerges when we explore the underlying concepts related to the topic in greater depth. Responses to our first question during individual interviews and focus groups with both professions indicate that there is no consensus on what this topic entails. Additionally, our study identified at least five different models representing concepts of internships on the master’s level. Regardless of the content of these models or the question which of them is most suitable, the diversity of concepts challenges the TPB-based results on barriers and facilitators.

In the first part of the study, the theory of planned behaviour used to identify factors that either inhibit or facilitate the involved parties in conducting internships to exhibit the target behaviour. This target behaviour was defined as conducting internships within a master's degree programme at a master's level. The intention was to create a taxonomy of these factors to develop a systematic guide for delivering the practical components of the programme at the master's level. However, the decision to use this method assumed a shared understanding of what constitutes the master's level.

The results from the focus groups in the second part of the study raise doubts about the interpretation of the first part's results. This second part suggests that the factors identified through TPB refer to a wide range of definitions of the target behaviour. The specific definition considered by interviewees in the first part remains unclear. Without a precise behavioural reference, the taxonomy of behaviour moderating factors—categorised into attitudes, social norms, and control beliefs—seems of little use. The second part of the study, meant to validate the first study's findings, ends up challenging them. Upon closer inspection, this issue might also apply to corresponding findings in the literature.

The reader might wonder about which of the five models is most suitable. First, we must consider that there might be other models due to potential selection bias and limited context variation. For instance, we discovered a study on the postgraduate midwifery programme at Lira University in Uganda [23]. This programme focuses on critical clinical competencies for master’s level midwives compared to bachelor’s, leading to better clinical outcomes for mothers and children. However, this model does not fit the Norwegian system.

Second, although some models derived from the data are clearly less mature than others, and at least the first two models (the pure practise model and the add-on practise model) are not well-balanced, we don’t feel equipped to rank them. We believe it’s crucial for professional leaders in practise, politics, and universities to agree on what master’s practise training should entail.

Third, we propose enhancing the discussion by focusing on the competencies and functions of a master in practise work, rather than on the theory versus practise debate. The functions of advanced practise nurses might offer guidance in this area [21, 22]. These functions include specialisation in clinical activity, coaching, consulting, collaboration, leadership, ethical decision-making, and research competence [22]. These functions also involve implementing change and challenging existing management and care structures to meet user needs or institutional demands [22]. The question remains: what should a master’s student learn in order to become capable of practising these functions effectively?

Our results reflect the situation within two Norwegian healthcare professional environments and may be applicable to other healthcare education master’s programmes in Norway. Although we have no evidence to compare our results with other countries participating in the Bologna Process or countries with comparable education frameworks and structures of academic degrees like the Australia's Australian Qualifications Framework (AQF) and the UK’s Framework for Higher Education Qualifications (FHEQ), we think that our particular research question might be of high relevance. The academisation of health professions is a global phenomenon. The difficulties to translate academic competencies into practise settings, which are characterised by tradition and pragmatism might be generic of nature. However, it is possible that our study illuminates a regional phenomenon. Compared to other European countries, the Norwegian health system may not sufficiently highlight differences. In our study, participants from both professions noted that master’s degree holders typically do not receive better salaries, privileges or other functions than health professionals without a master’s degree. It seems national policy expects everyone to complete a master’s degree. However, there is less discussion about how healthcare provided at a master’s level differs from healthcare without. These observations suggest more emphasis on the role of cultural habits related to the communication and handling of variation regarding performance and quality in social environments. It is clear that a culture that negates social differences is not a good environment to facilitate innovation and quality improvement such as intended by the lifting of health professions on an academic level.

## Conclusions

Our study revealed a lack of awareness and agreement on what is making internships to be part of a master’s training and how master’s training in practise should be shaped to facilitate this learning. This refers to two quite different Norwegian health education programmes. The widespread conceptualisation is making it difficult to interpret perceived challenges in the realisation of internships which have been collected from the involved participants. The implementation of the master's level with the joint efforts of all those involved is doomed to failure as long as it is not clear what exactly is to be implemented. More research is needed to investigate generalisability of our results to other programmes and other countries.

## Supplementary Information

Supplementary Information 1.

## Data Availability

Data are available from the last author.
