# Supplementary material for: How the master’s level is implemented in internships within master’s programmes — exploring the views of students, clinicians, and educators in midwifery and public health nursing
Source: BMC Med Educ. 2024 Dec 19;24:1496. doi: 10.1186/s12909-024-06461-4 (PMC11657649; doi:10.1186/s12909-024-06461-4)
Supplement: Supplementary file 1 — Supplementary Information 1. [file 12909_2024_6461_MOESM1_ESM.pdf]

Master internship project interview guide

Interview guide and structured documentation sheet: The sheet is supposed to be used in preparation of, during and after the interview. During the interview, it might be useful to hold an eye on the sheet to be reminded of the TPB (theory of planned behaviour) categories. It is not recommended to fill the sheet during the interview. Meaningful elements must not be rejected simply because they are difficult to categorise. Keep them by copying them into empty textboxes, for later categorization.

You are asked to collect information about:

**Role description**
Which role and function does the interviewee have? Important to explore the interviewee's function in arranging internships for master's students?

**Operationalization of the master's level within internships**
What does master's level in internships mean in your opinion?

Which barriers and facilitators related to realizing the master's level in internships are mentioned? Barriers can be framed as facilitators and the other way round, e.g., reports of coping a challenge can indicate a barrier, if the coping skills are lacking.

**TPB form** for documentation of barriers and facilitators towards conduct of internship on master's level			
category		definition	Utterance (example)
Attitude	Expectations	Expectation related to consequences of customizing the internship for master's level.	*(If we would do that, we would need to spend much more time for those students)*<br>…<br>…<br>…<br>…<br>…
	Value	Individual appraisal of the value in the consequences above.	*(This would mean a catastrophe as time is under pressure already now)*<br>…<br>…<br>…<br>…<br>…
Subjective social norm:	expectations	Expectations about what important other do expect the interviewee to do	*(We do feel pressed by the university)*<br>…<br>…<br>…<br>…<br>…
	Values	Willingness to comply with expectations of important others.	*(But we do not care so much).*<br>…<br>…<br>…<br>…<br>…
Control believes	Expectations	Expectations about coping of challenges the interviewee is exposed to when internships are supposed to be customized for master's level	*(I am a skilled practitioner, however, not able to meet such students on an academical level)*<br>…<br>…<br>…<br>…
	Values	Utterance about how much the interviewee cares about coping in this context	*(This means big stress to me)*<br>…<br>…<br>…<br>…
